# Exercise and cardiac rehabilitation after LVAD implantation

**DOI:** 10.1007/s10741-024-10477-9

**Published:** 2024-12-27

**Authors:** Emily Newman, Yevgeniy Brailovsky, Indranee Rajapreyar

**Affiliations:** 1 Division of Cardiology, Jefferson Heart Institute, Sidney Kimmel School of Medicine, Thomas Jefferson University, 833 Chestnut Street, Suite 630, Philadelphia, PA 19107 USA; 2 Division of Cardiology, Tufts Medical Center, Boston, MA USA

**Keywords:** Left ventricular assist devices, Cardiopulmonary exercise testing, End-stage heart failure

## Abstract

Left ventricular assist devices (LVAD) have improved mortality and quality of life for patients with end-stage heart failure by providing an alternative to cardiac transplant or as a bridge to transplantation. The improvement in functional capacity however is minimal to modest depending on the right ventricular function, optimal hemodynamics on LVAD therapy, and comorbidities. There is improvement in submaximal exercise capacity but improvement in peak aerobic capacity is limited. Exercise capacity can be improved by referral to cardiac rehabilitation after LVAD implantation. Cardiac rehabilitation is safe and effective with improvement in functional capacity, and decrease in mortality and heart failure hospitalizations. Cardiopulmonary exercise testing (CPET) is a specialized exercise stress test that measures gas exchange during exercise to determine a variety of variables that have been shown to be predictive of mortality in patients undergoing cardiac transplant. CPET is valuable for prognostication and is a predictor of adverse outcomes, including right heart failure in the immediate post-LVAD implantation period, long-term mortality. CPET is an additional testing that can aid in the decision making for LVAD explantation or decommissioning.

## Introduction

Left ventricular assist devices (LVAD) have changed the landscape of end-stage heart failure by providing an alternative to cardiac transplantation either as bridge to transplant or as destination therapy. With improvements in technology, hemocompatibility-related adverse events like pump thrombosis and stroke have decreased. LVAD implantation is associated with improved short- and long-term survival and quality of life for end-stage systolic heart failure patients [1, 2]. The improvement in exercise performance after LVAD implantation is variable and modest [2]. Cardiopulmonary exercise testing (CPET) has shown to have prognostic value in patients with heart failure, but its prognostic ability in LVAD patients is limited in the literature.

In healthy individuals, the physiologic changes during exercise include coordinated responses from skeletal muscle, respiratory, and cardiovascular systems. The cardiovascular responses to aerobic exercise result in increased stroke volume, heart rate (HR), and cardiac contractility to augment cardiac output. Exercise intolerance in patients with heart failure or LVADs is multifactorial including impaired respiratory gas exchange, myocardial relaxation and contractile dysfunction, skeletal muscle abnormalities, anemia, chronotropic incompetence, and valvular heart disease [3].

CPET is a specialized exercise stress test used to determine maximum exercise capacity by measuring gas exchange to determine O2 uptake (**V̇**O2), carbon dioxide production (**V̇**CO2), and ventilation (Ve) [3]. These variables can then be used to derive further assessments of exercise capacity including peak **V̇**O2, which incorporates heart rate, stroke volume, and oxygen extraction using the Fick principle. Peak **V̇**O2 has been used as a predictor of mortality in patients with HFrEF, and for risk stratification of end-stage heart failure patients for cardiac transplantation. A landmark study by Mancini et al. showed peak **V̇**O2 < 14 ml/kg/min in patients with severe left ventricular systolic dysfunction was associated with decreased 1-year survival [4]. In the presence of beta-blockers, the peak **V̇**O2 < 12 ml/kg/min is used because of improved survival with beta-blockers [5, 6]. Younger patients and those at the extremes of BMI should have peak **V̇**O2 compared to predicted values, and < 50% indicates poor prognosis. Other CPET variables that are of prognostic value include ventilatory efficiency, exercise oscillatory ventilation, and hemodynamic response to exercise. Ventilatory efficiency is defined by the slope formed by Ve/**V̇**CO2 and values > 34–36 have prognostic significance. CPET can be combined with invasive hemodynamic monitoring for more precise and integrated measurements of hemodynamics and exercise performance measures during rest and exercise [3]. CPET can also be combined with noninvasive imaging, such as echocardiography for further information on diastolic function, pulmonary hypertension, and valvular heart dysfunction with exercise [7].

This article will review available literature on assessment of functional capacity after LVAD, utility of CPET for prognostication, and utility of cardiac rehabilitation after LVAD implantation.

## Left ventricular assist device physiology at rest

Pulsatile left ventricular devices (pLVAD) were replaced by continuous-flow left ventricular devices (cfLVAD) due to device durability. HeartMate III is the only continuous flow device (centrifugal) that is currently being implanted in the USA due to the superior hemocompatibility profile compared to other cfLVAD’s.

At rest, cardiac output in a cfLVAD patient is combined flow through the LVAD and variable aortic valve opening. The pressure differential between the inlet and outlet (mean arterial pressure) determines flow through the cfLVAD. Right ventricular function in a cfLVAD patient contributes to left ventricular preload and cardiac output.

## LVAD physiology with exercise

**Fig. 1:**
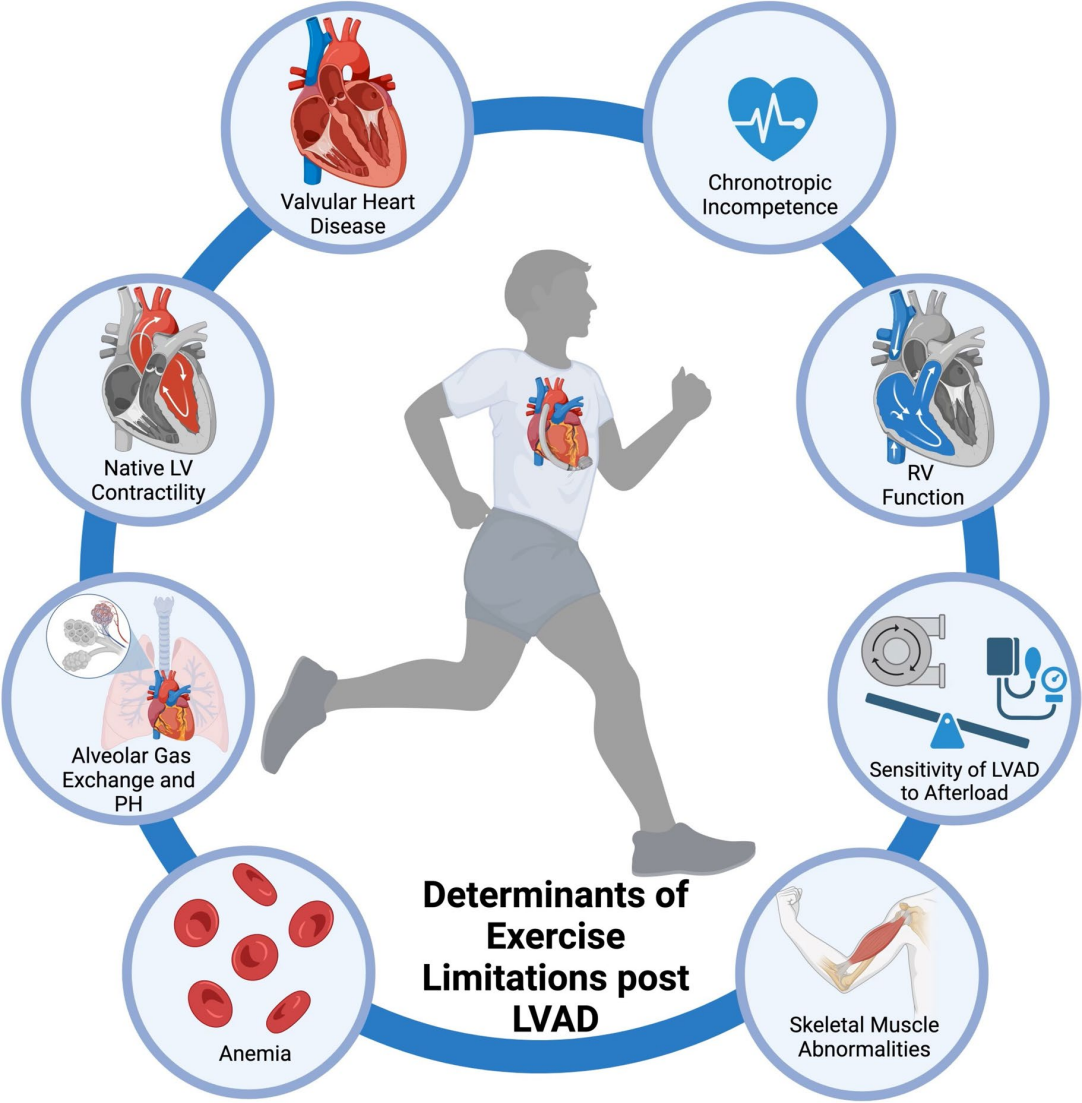
Determinants of exercise limitations after LVAD implantation

Cardiovascular response during aerobic exercise with cfLVAD is dependent on right ventricular function, sensitivity of the device to afterload, chronotropic incompetence, and native myocardial contractility. Other factors include anemia, skeletal muscle abnormalities, and impaired alveolar gas exchange due to chronic heart failure (Fig. 1) [8].

At a set LVAD speed, decongested state and normal cardiac output at rest are acceptable to prevent rehospitalizations due to heart failure. However, exercise tolerance at a set LVAD speed depends on residual native left ventricular function and exercise-induced hypertension. In LVAD patients undergoing maximal and submaximal exercise testing, max LVAD pump output was only 1 l and did not correlate with peak **V̇**O2. However, aortic valve opening and increase in HR correlated with peak aerobic capacity highlighting the importance of residual native LV contractility [9]. Noor et al. reported the findings of maximal exercise test in cfLVAD patients with left ventricular ejection fraction (LVEF) either less or more than 40% and at set or reduced LVAD speed. cfLVAD patients with LVEF < 40% had lower peak **V̇**O2 at set speed which decreased further by 2.5 ml/kg/min with speed reduction but patients with LVEF > 40% had higher peak **V̇**O2 at set speed which did not change with speed reduction. Patients with LVEF < 40% also had ventilatory inefficiency at baseline set speed which increased further with speed reduction [10]. Cardiopulmonary stress testing with cfLVAD pump speed increase every 2 min improved maximum aerobic capacity compared to fixed pump speed by 9.2% [11].The ventilatory efficiency parameters also improved in the LVAD speed increase group which may be due to improved left ventricular unloading during exercise [11].

Improved oxygen uptake was dependent on native left ventricular systolic function and chronotropic competence in a subset of patients who underwent CPET approximately a year after LVAD implantation [12]. Camboni et al. reported impaired exercise capacity and elevated pulmonary artery and left atrial pressures in LVAD patients with baseline fixed speed to allow aortic valve opening and pulsatility [13]. In a study that evaluated invasive hemodynamics and peak exercise capacity in patients supported on axial-flow and centrifugal pumps, peak **V̇**O2 was dependent on maximal heart rate, native LV contractility, and peripheral oxygen extraction [14]. The peak exercise capacity was dependent on native LV contractility as demonstrated by increase in Fick cardiac output with minimal contribution from LVAD pump flow. Exercise in a cfLVAD patient unmasked the hemodynamic profile of elevated pulmonary artery pressures and pulmonary capillary wedge pressures seen in patients with heart failure with reduced ejection fraction [14–16]. This underscores the importance of compensated hemodynamics at rest with cfLVAD but inadequate support with exercise. Current-generation cfLVAD do not have automated algorithms for speed modulation during exercise. However, speed adjustment during exercise without monitoring can result in suction events and ventricular arrhythmias.

## Exercise capacity after continuous-flow LVAD

Continuous-flow LVAD implantation does not improve peak oxygen uptake during symptom limited exercise [12, 14, 16–20]. Seventy-two percent of patients had peak **V̇**O2 < 14 ml/kg/min after cfLVAD implantation [18]. Submaximal parameters of cardiopulmonary stress testing, like VE/**V̇**CO2 and **V̇**O2 at anaerobic threshold, remain impaired after cfLVAD support [14]. Felix et al. showed improvements in maximal workload and peak oxygen uptake by 9% and 7%, respectively, at 1 year after cfLVAD implantation. Maximal heart rate at exercise, younger age, and lower body mass index (BMI) were predictors of improvement in exercise capacity. Patients with ischemic cardiomyopathy had a lower peak oxygen uptake compared to nonischemic dilated cardiomyopathy [21].

There were improvements in submaximal stress testing which was evaluated by the 6-min walk test [12, 17, 19, 22]. The maximum improvement in 6-min walk distance was seen in the 3 months post cfVLAD implantation and was unchanged at 2-year follow-up [23].

## Right ventricular dysfunction and exercise capacity after cfLVAD

RV dysfunction contributes to morbidity and mortality after LVAD insertion. CPET can help prognosticate patients at risk for postoperative RV dysfunction. VE/**V̇**CO2 slope has been proposed as a predictor of postoperative RV failure and overall mortality. VE/**V̇**CO2 slope > 36 is associated with reduced TAPSE and reduced RV EF in the general heart failure population. Evaluation of 70 patients with CPET preformed prior to LVAD implantation demonstrated that VE/**V̇**CO2 > 36 had higher mortality than patients with lower VE/**V̇**CO2 (30.2% vs 7.4%). VE/**V̇**CO2 > 36 was also associated with acute severe or severe right heart failure. Further analysis suggests a VE/**V̇**CO2 of 50 predicts need for RV mechanical support or death secondary to RV failure [24].

Early and late right heart failure after LVAD implantation can limit aerobic capacity. Studies evaluating right ventricular dysfunction and the contributions to exercise capacity after cfLVAD implantation have yielded conflicting results. Presence of tricuspid regurgitation, a surrogate for right ventricular function after cfLVAD implantation, correlated with impaired p**V̇**O2 [22]. Preserved right ventricular function as measured by tricuspid annular plane systolic excursion (TAPSE) > 13 mm after cfLVAD implantation predicted maximum aerobic capacity after cfLVAD [25]. Right ventricular contractility in LVAD patients improved with submaximal exercise but no further increases in contractility were noted during maximal exercise highlighting the lack of RV contractile reserve as another contributor to reduced exercise capacity [16].

## CPET for prognostication after LVAD

CPET has been used to evaluate patients for LVAD placement although much of the available literature includes retrospective studies evaluating those patients who had CPET preformed prior to LVAD placement. This does bias against those patients who are too unstable to undergo this type of testing prior to LVAD consideration.

A retrospective study evaluating CPET before and after LVAD implantation was able to associate several variables with 1-, 3-, and 5-year mortality. No CPET variable was statistically significantly associated with post-LVAD ICU length of stay, 30-day readmission rates, or 90-day mortality. **V̇**O2 max > 9.7 ml/kg/m^2^, oxygen uptake efficiency slope (OUES) > 951, vE/**V̇**CO2 slope > 50.17, vE/**V̇**CO2 min > 47.45, and vE/**V̇**CO2max > 61.28 were significantly associated with 5-year survival. **V̇**O2 max, OUES, VE/**V̇**CO2 slope, and VE/**V̇**CO2 were associated with 3-year survival and **V̇**O2max and OUES were associated with 1-year post-implantation survival. In repeat CPET after LVAD implantation, only **V̇**O2 max > 11.8 was the independent predictor of 5-year survival and **V̇**O2 max > 11.8 and VE/CO2 max > 57.61 predicted 3-year survival [26].

In addition to peak **V̇**O2, the change in blood pressure during CPET has prognostic value for post-LVAD implantation mortality. A study of 156 patients who had CPET prior to LVAD placement was stratified by those whose blood pressure increased with exercise and those whose blood pressure decreased despite similar resting blood pressures. Patients with decrease in blood pressure during CPET had increased mortality during initial admission, at 1 month and 3 months. Of note, peak **V̇**O2 was significantly lower in the hypotensive group but other CPET variables were not statistically significant among the groups [27].

Peak **V̇**O2 retains it prognostic value after LVAD implantation. Patients with lower p**V̇**O2 had higher mortality and higher hospital readmission rates. In the PRO-VAD study, patients had CPET within 1 year of implant and those patients with **V̇**O2 > 14 ml/kg/min (no beta blocker) or > 12 ml/kg/min (on beta blockers) had improved survival at 1 year. This suggests that these cutoffs can be used to identify LVAD patients in whom transplant can safely be deferred, akin to those heart failure patients who do not have LVAD [28].

## CPET use in LVAD decommissioning

Patients who recover cardiac function after LVAD implantation may be candidates for LVAD decommissioning. There is great variability in criteria for LVAD decommissioning and CPET can be used to help to guide that decision. Some protocols use peak **V̇**O2 > 16 ml/kg/min and Ve/**V̇**CO2 slope < 34 with the absence of periodic breath/exercise oscillatory ventilation during testing at low LVAD speeds as indications of LV recovery and appropriateness for explantation [29]. Another protocol suggests explantation criteria of LVEF > 45%, LV end diastolic dimension < 6 mm, PCWP < 12 mmHg, CI > 2.8 with peak **V̇**O2 > 16 ml/kg/min, or Ve/**V̇**CO2 slope < 34 at low LVAD speeds [30]. An evaluation of 33 patients with CPET after LVAD implant identified a combination of three CPET variables (maximum load ≥ 51 W, Ve/**V̇**CO2 slope < 34, and p**V̇**O2 > 12.8 ml/kg/min) predicted successful LVAD explantation [31]. A small study evaluated combining the use of CPET with echocardiogram to evaluate candidates for LVAD explantation. Patients had CPET with echocardiogram performed at optimized LVAD speed and repeat testing within 14 days at the lowest possible LVAD speed. No significant change in CPET or echocardiogram at baseline vs lowest LVAD speeds was supportive of LV recovery and indicated who would tolerate device explant. Two patients had LVAD explantation using this protocol and remained event free after more than a year [7].

## Cardiac rehabilitation post cfVAD

Rehabilitation after cfLVAD implantation can be divided into two phases: inpatient rehabilitation versus home physical therapy and cardiac rehabilitation.

Each phase of rehabilitation care is aimed at improving functional capacity to perform activities of daily living, mobility, dexterity in handling LVAD equipment, psychosocial support, and cognition. There is continued medical optimization of residual heart failure symptoms and combined echocardiographic and hemodynamic optimization on LVAD support. Criteria are established to terminate exercise therapy sessions in a patient with an LVAD and include sustained decrease in LVAD flow < 3 l/min, symptomatic hypotension (mean arterial pressure < 70 mmHg), exertional dyspnea or chest pain, ventricular arrhythmias, hypoxia (oxygen desaturation below 90%), and decompensated heart failure [32, 33].

Patients after LVAD implantation often require inpatient rehabilitation due to frailty, deconditioning, and continued heart failure [34]. The goals of acute inpatient rehabilitation are to improve Functional Independence Measure (FIM), which encompasses 6 domains including self-care, transfers, locomotion, sphincter control, communication, and social cognition. Acute inpatient rehabilitation has shown to improve FIM in patients after LVAD implantation in patients who are debilitated [34–38].

Exercise capacity is limited after LVAD implantation and is limited to low-intensity workload with impaired peak oxygen uptake after LVAD implantation similar to patients with HFrEF [20]. Safety and efficacy of cardiac rehabilitation (CR) after LVAD is well established [39]. CR after LVAD implantation is associated with decreased mortality and hospitalization [27]. The objective of the CR program is to improve cardiorespiratory conditioning via exercise training, psychosocial support, medical therapy, and optimization [40, 41]. Patients who completed CR had improved survival at 1 year (96%) compared to patients who declined to participate or were too sick to enroll in CR. Patients who were considered sick to enroll in CR had increased hospitalizations and 2.85-fold increase for mortality at 1 year [40]. Exercise capacity measured by peak oxygen uptake increased significantly after CR albeit not to normal due to several factors discussed in the LVAD physiology with exercise and exercise with continuous-flow LVAD sections [39]. In a single-center study that randomized patients to CR and usual care without exercise prescription, patients randomized to CR with a total of 18 sessions over 6 weeks had 10% improvement in functional capacity, muscle strength, and health status as measured by Kansas City Cardiomyopathy Questionnaire (KCCQ) [42].

**Fig. 2:**
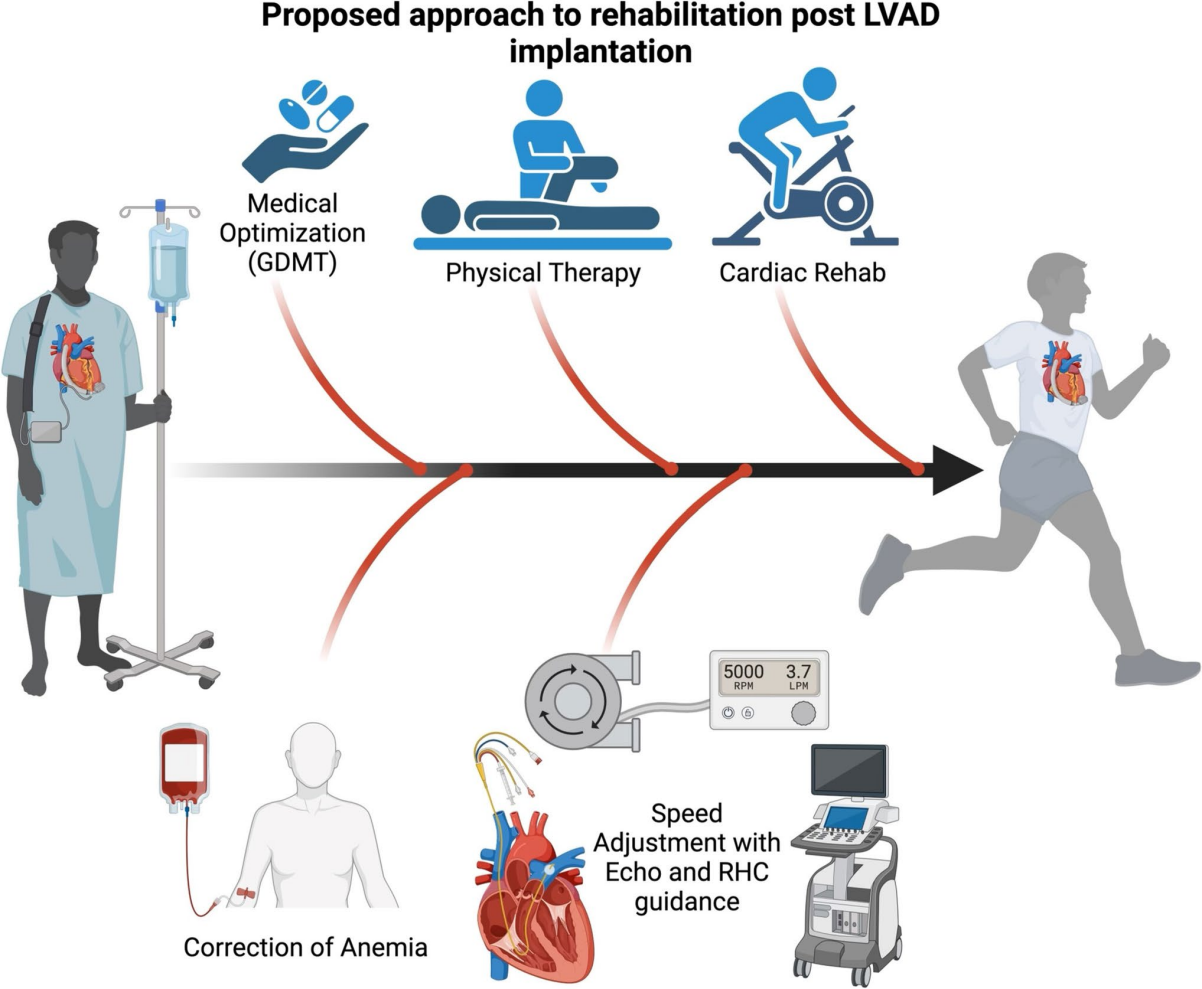
Proposed approach to rehabilitation after LVAD implantation

Exercise prescription should be personalized with established goals based on medical history. An ongoing collaboration between the heart failure cardiologists and the rehabilitation center will ensure success in this complex group of patients (Fig. 2).

## Health status assessment

Health-related quality of life incorporates a patient’s physical symptoms of disease with the emotional and psychosocial impacts of the illness and illness care. Repeat hospitalizations or adverse effects from the LVAD impact quality of life and health status as does the learning to live with the new implant. There are several validated questionnaires, including the Kansas City Cardiomyopathy Questionnaire (KCCQ) and the Minneapolis Living with Heart Failure Questionnaire (MLHFQ) which are commonly used to evaluate how heart failure therapies affect quality of life [43, 44]. Data using these questionnaires in LVAD patients is limited, although a study using an INTERMACS registry on health status after HM2 placement found that that 7.3% of patients reported continued poor health-related quality of life (KCCQ < 45) at 1 year after implant of LVAD for destination therapy [45, 46]. More studies are needed to define the therapeutic effects on quality of life in LVAD patients.

## Conclusions

The evolution of LVAD device technology has improved survival and health-related quality of life, and decreased hemocompatibility adverse events. The next phase of improving life with an LVAD is to improve functional capacity and decrease hospitalizations due to heart failure. CPET may prove instrumental in guiding care including prognostication of risk prior to and after LVAD implantation, including identifying those at increased risk for RV failure. Patients have significant impairments in exercise capacity after LVAD implantation which highlight the importance of continued rehabilitation. This often involves inpatient rehabilitation programs followed by cardiac rehabilitation, which have shown to improve functional capacity for patients with LVADs. The rehabilitation period after LVAD implantation represents another area where CPET could provide useful clinical information to monitor patients. Ultimately, more research, including prospective studies, is required to determine the most appropriate use of CPET and rehabilitation practices for this advanced heart failure population.
